# Assessing the Importance of Domestic Vaccine Manufacturing Centers: An Overview of Immunization Programs, Vaccine Manufacture, and Distribution

**DOI:** 10.3389/fimmu.2018.00026

**Published:** 2018-01-18

**Authors:** Emma Rey-Jurado, Felipe Tapia, Natalia Muñoz-Durango, Margarita K. Lay, Leandro J. Carreño, Claudia A. Riedel, Susan M. Bueno, Yvonne Genzel, Alexis M. Kalergis

**Affiliations:** ^1^Millennium Institute on Immunology and Immunotherapy, Departamento de Genética Molecular y Microbiología, Facultad de Ciencias Biológicas, Pontificia Universidad Católica de Chile, Santiago, Chile; ^2^Max Planck Institute for Dynamics of Complex Technical Systems, Magdeburg, Germany; ^3^Departamento de Biotecnología, Facultad de Ciencias del Mar y Recursos Biológicos, Universidad de Antofagasta, Antofagasta, Chile; ^4^Millennium Institute on Immunology and Immunotherapy, Programa de Inmunología, Instituto de Ciencias Biomédicas, Facultad de Medicina, Universidad de Chile, Santiago, Chile; ^5^Millennium Institute on Immunology and Immunotherapy, Departamento de Ciencias Biológicas, Facultad de Ciencias Biológicas y Facultad de Medicina, Universidad Andrés Bello, Santiago, Chile; ^6^Departamento de Endocrinología, Facultad de Medicina, Pontificia Universidad Católica de Chile, Santiago, Chile

**Keywords:** vaccine manufacturing, immunization programs, vaccine distribution, vaccine shortages, good manufacturing practices

## Abstract

Vaccines have significantly reduced the detrimental effects of numerous human infectious diseases worldwide, helped to reduce drastically child mortality rates and even achieved eradication of major pathogens, such as smallpox. These achievements have been possible due to a dedicated effort for vaccine research and development, as well as an effective transfer of these vaccines to public health care systems globally. Either public or private institutions have committed to developing and manufacturing vaccines for local or international population supply. However, current vaccine manufacturers worldwide might not be able to guarantee sufficient vaccine supplies for all nations when epidemics or pandemics events could take place. Currently, different countries produce their own vaccine supplies under Good Manufacturing Practices, which include the USA, Canada, China, India, some nations in Europe and South America, such as Germany, the Netherlands, Italy, France, Argentina, and Brazil, respectively. Here, we discuss some of the vaccine programs and manufacturing capacities, comparing the current models of vaccine management between industrialized and developing countries. Because local vaccine production undoubtedly provides significant benefits for the respective population, the manufacture capacity of these prophylactic products should be included in every country as a matter of national safety.

## Introduction

The incidence of numerous infectious diseases that are life threatening to humans has drastically declined since the development of safe and effective vaccines and the implementation of global vaccination programs worldwide. In fact, the variola virus, which caused smallpox disease that killed millions of individuals throughout history, was successfully eradicated from Earth during the 1980s (1), due to a worldwide immunization campaign against this major pathogen. Moreover, poliovirus, which severely affects the health of children with lifelong disabling consequences, has almost been eradicated from the world. Since 1999 very few cases of polio disease have been reported, probably due to two of the three poliovirus types. Indeed, the goal of the World Health Organization (WHO) is to achieve the eradication of polio during 2018. Therefore, millions of human lives have been saved by means of the implementation of national immunization programs in all countries, and the demand for new prophylactics to protect against infectious diseases is constantly growing. Although vaccine manufacturing is usually associated with biopharmaceutical companies, some public and academic institutions also produce these prophylactic formulations (2). Despite the existence of those manufacturers aiming at increasing vaccine availability, shortage of these products has taken place several times causing that not enough doses were available in some countries.

In this article, we attempt to comprehensively discuss the WHO current recommendations for routine immunization and some of the national immunization programs. Further, we associate such vaccination programs to the global vaccine manufacture and distribution capabilities, focusing in some industrialized and developing countries. The comparison between these two types of nations was done to point out key management differences among them, when aiming at guaranteeing prophylaxis against serious infectious diseases in their populations. In addition, we also examined the dependency on foreign vaccine supply of some countries, classifying them according to their capacity to supply the local demand with domestic facilities or *via* importation from other states.

## Vaccines Currently Recommended by the WHO

**Figure 1:**
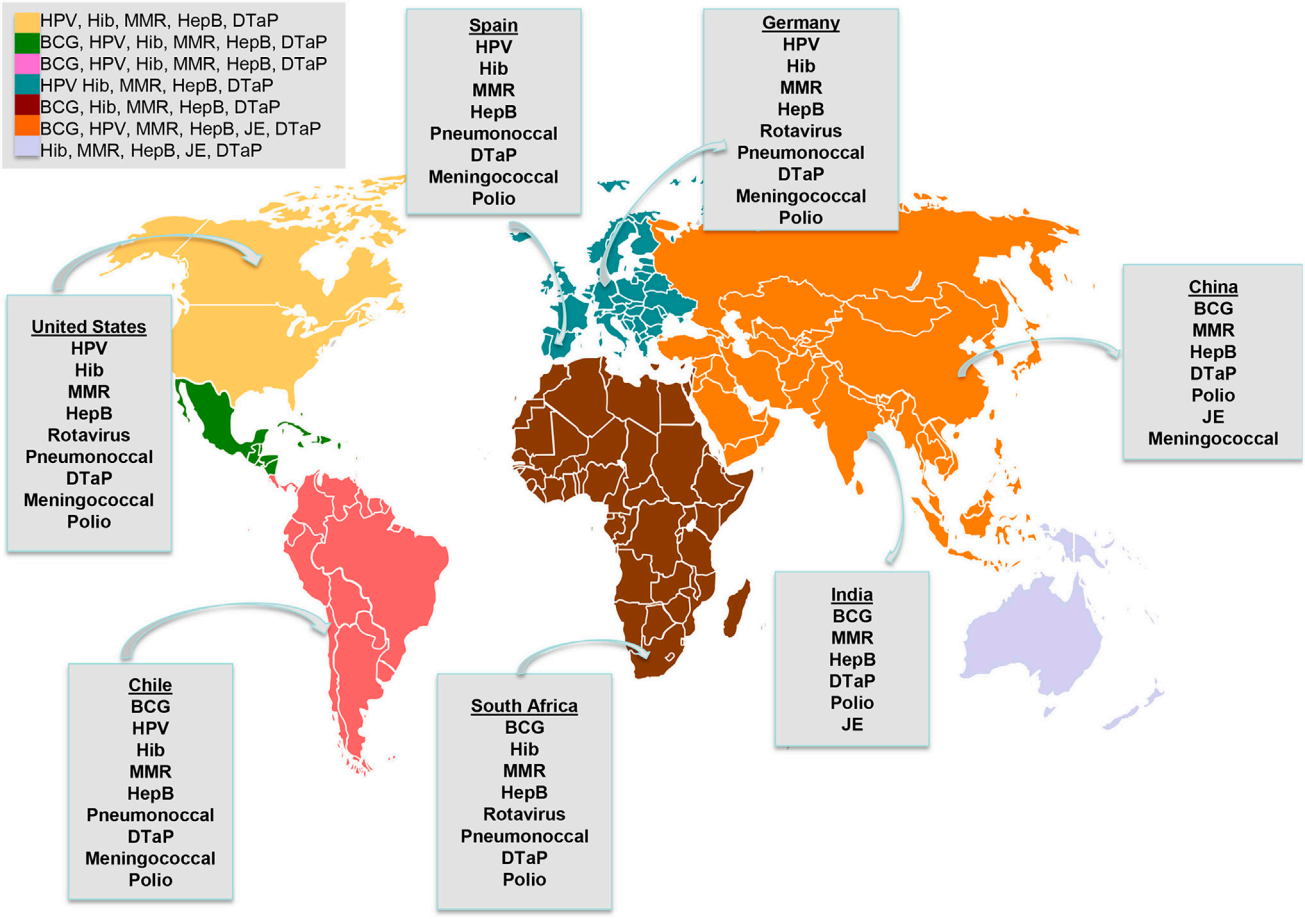
Immunization programs around the world. Vaccines funded by national governments and included in national immunization programs by continent and regional examples. BCG, bacille Calmette–Guerin; HepB, hepatitis B virus; DTaP combines protection against diphtheria, tetanus, and pertussis; MMR, combines mumps, measles and rubella; Hib, *Haemophilus influenzae* type B; HPV, human papillomavirus; JE, Japanese encephalitis live vaccine. Exemptions: BCG is given in some countries of Europe. HPV is given in some countries of Africa.

According to the WHO, children should be immunized with bacille Calmette–Guerin (BCG), diphtheria-tetanus-acellular pertussis (DTaP), MMR (combines Mumps, Measles, and Rubella), and vaccines to prevent Hepatitis B, poliovirus, *Haemophilus influenza*e type B (Hib), several serotypes of *Streptococcus pneumoniae*, rotavirus, and papillomavirus (3). In addition to these vaccines for children, the influenza vaccine is also recommended to be administered in certain susceptible groups, such as pregnant women, healthcare workers, children aged 6–59 months and the elderly (>65 years old) (3). Furthermore, the coverage of routine Expanded Program on Immunization (EPI), which includes vaccines against tuberculosis (TB), diphtheria, tetanus, and pertussis, polio and measles, varies from country to country (Figure 1).

### Vaccination for Poliomyelitis: An Example of a Nearly Eradicated Disease

Although poliomyelitis cases decreased greatly in 1988, 74 cases of this disease were reported in 2015. The majority of them occurred in Pakistan and in Afghanistan. Therefore, the goal proposed by the WHO is to eradicate poliomyelitis by 2018. Poliomyelitis is an infection caused by poliovirus that affects the human nervous system (4). The trivalent attenuated oral polio vaccine (tOPV), which includes the types 1, 2, and 3, has been used since the beginning of the 1960s. However, due to the polio type 2 vaccine components were pointed out as the infectious source leading to a large number of cases of vaccine-derived polioviruses, global initiatives have suggested to switch from the trivalent to a bivalent polio vaccine. Such vaccine only includes type 1 and 3 viruses (5). Interestingly, the wild type poliovirus type 2 has not been reported since 1999 and was declared eradicated in September 2015. Besides, the poliovirus type 3 has not been detected since 2012 and the poliovirus type 1 is likely the only strain remaining in circulation.

As an additional effort to keep population protected against all types of poliovirus during the eradication program, the WHO instructed to include at least one dose of the inactivated polio vaccine (IPV) in the sequential shift from the tOPV toward the dOPV (6). The IPV is composed by the three types of poliovirus, which is intramuscularly administered. Clinical trials in children have shown that this vaccine is an excellent booster and capable of enhancing the mucosal immune response in primed subjects (4, 7).The future goal is to shift from dOPV to IPV at the time when type 1 and 3 polioviruses were eradicated.

### Vaccination for Respiratory Diseases: TB, Pneumonia, and Influenza

A different vaccine type, the BCG vaccine, has been used in over a billion people since 1921 to prevent TB (8). Although not able to induce a strong protective immunity in adults, the BCG vaccine has been shown to protect against meningitis TB disease in children (9). However, the BCG vaccine currently is not utilized in children from countries with low rates of TB incidence, such as the USA, Spain, Australia, Norway, Canada, and England (10). In those countries, the BCG vaccine is only recommended for those children showing a negative tuberculin skin test and that are continually exposed to adults with untreated or ineffectively treated for TB disease. Further, BCG vaccination is also recommended for health care workers in settings of frequent exposure to TB patients (11). Furthermore, because the BCG vaccine derives from attenuated bacteria passaged in the 1960s, the large number of passages affecting the banks available today has led to multiple genetic changes in the bacilli. Several studies supported the notion that this genetic divergence could be responsible for the variant protective capacity against TB shown by the various BCG vaccine strains (8, 12). Thus, an efficient BCG vaccine that provides full protection is still required. The major BCG manufacturers prequalified by the WHO are the Staten Serum Institute (Denmark), Serum Institute of India Ltd., Japan BCG Laboratory, and Intervax Ltd. (Canada). In addition, some Asian and Eastern-European countries possess their own locally-produced BCG vaccine, such as China (China National Biotec Group), Serbia (Torlak Institute), and Vietnam (IVAC) (8).

Bacteria-caused pneumonia, due to infection with various serotypes of *S. pneumoniae* (Pneumococcal disease) and Hib display a high rate of morbidity and mortality worldwide, although nowadays, the majority of the deaths take place in sub-Saharan Africa and Asia (13). Both pneumococcal and Hib vaccines are recommended by the WHO (3). However, not all countries include these vaccines in their national immunization programs and, for instance, the public health systems of some South Asian countries do not use them at all (Figure 1). Thus, whereas these vaccines were introduced in the 1990s in most industrialized countries, still these prophylactics are not funded by public health systems in some developing countries, such as South Asian nations. Consequently, still 18/100,000 and 26/10,000 cases of Hib were reported in children younger than 5 years old in Vietnam and in China, respectively. To handle these high incidence rates, organizations including the Global Alliance for Vaccine and Immunization (GAVI) and the United Nations International Children’s Emergency Fund have financed pneumococcal and Hib vaccines to provide coverage for developing countries (14). Several studies conclusively have supported the notion that public health systems should add these vaccines to their national immunization programs with their own funding, in every developing country. Thus, adopting these measures, the incidence of these major infectious diseases could be reduced (15, 16). Further, some GAVI-supported countries experienced a transition from GAVI-derived support to a fully self-financed Hib vaccination program. Thereby, strategic immunization plans are required to provide vaccines to their population (17).

Viral respiratory diseases generated by the influenza virus causes low rates of mortality but high rates of morbidity worldwide every year (18–20). This seasonal disease is mainly caused by two types of influenza viruses: A and B (21). The influenza A virus displays a high rate of variation causing frequent antigenic changes, in a process known as antigen drift. For this reason, the influenza vaccine confers only limited-time protection (up to 2 years) and it is necessary to reformulate and manufacture new influenza vaccines every year. Influenza vaccination is recommended by the WHO for high-risk individuals, including children, pregnant women, healthcare workers, the elderly and individuals suffering from chronic conditions, such as asthma, diabetes and heart disease (3). Further, organizations such as the American Academy of Pediatrics recommend the seasonal influenza vaccination for children of 6 months and older (22). However, the coverage of this vaccine still remains low despite the influenza vaccination strategies, including government involvement and national programs (23). Importantly, pandemic influenza H1N1 emerged in 2009, affecting mainly children and the elderly. The global number of deaths during the first 12 months of virus circulation was reported from 151,700 to 575,400 people (24). Moreover, the older age groups presented higher severity in post-pandemic influenza outbreaks (25).

### Vaccination to Prevent Diphtheria, Tetanus, and Whopping Cough

Another vaccine of global relevance is the DTaP (14). This vaccine protects against three severe infectious diseases: diphtheria, tetanus, and pertussis. First, diphtheria is caused by *Corynebacterium diphtheria*, which produces pharyngeal infection, myocarditis, polyneuropathy, and systemic toxicity (26). Second, tetanus is caused by *Clostridium tetani* and the typical symptoms include muscle spam and contraction (26). Finally, pertussis, also known as whopping cough, is caused by *Bortedella pertussis*, which can produce loss of weight, subconjunctival hemorrhages, and syncope (26). Currently these three diseases circulate in the population worldwide and the highest rates are observed in children from countries with low vaccination coverage, especially in developing countries (27–29). However, and despite high vaccination coverage, several outbreaks have recently been reported in industrialized countries (30). For instance, an outbreak in the USA was reported in 2012, resulting in 48,277 cases of pertussis (31). According to the United States Center for Disease Control and Prevention (CDC), DTaP protects from whopping cough in 7 out of 10 vaccinated subjects, while it efficiently protects against the severe illness. In fact, the introduction of DTaP vaccine in the USA reduced from 100,000 to 32,000 cases of pertussis per year. Despite these good results, DTaP could fail to provide long-lasting protection in humans (31). It is important to indicate that the WHO estimates that there still are about 16 million cases of pertussis and 30,000 of diphtheria per year worldwide, being the highest rates in India (32). Therefore, these infections are still an important public health burden that requires close monitoring.

### Vaccination to Prevent Cervical Cancer

The nine-valent human papillomavirus vaccine (HPV) is recommended for routine vaccination of girls at age of 9 or 10 years old to confer protection against cervical cancer caused by the HPV (33). This new vaccine is significantly more expensive as compared to the other vaccines. Thereby, although it is highly recommended vaccine, not all children are being immunized to prevent this cancer (33). Despite the fact that the first HPV vaccine was available in 2006, today only two biopharmaceutical companies manufacture this vaccine (33). A study performed in France showed 95.93% effectiveness for the HPV vaccine in sexually active young women (34). Despite such effectiveness, a strong parent refusal remains in several countries to vaccinate children against HPV due to safety and effectiveness concerns, as reported in a survey in the USA (35).

### Vaccination to Prevent Diarrheal Diseases

Another recent vaccine included in the immunization programs of several industrialized and developing countries is the one to prevent rotavirus-infections (3). This virus is one the most common causes of severe gastroenteritis with diarrhea-related hospitalizations in children worldwide, which shows in particular high mortality rates in developing countries (36). The WHO has recommended that this vaccine should be included in all national immunization programs, being strongly recommended for countries showing a high mortality rate in children under 5 years old due to severe dehydrating diarrhea (37). Nowadays, an increasing number of countries, such as the USA and Germany have incorporated the rotavirus vaccine in their national immunization programs. A meta-analysis conducted on individuals of Europe, North America and Latin America showed that this vaccine has an efficacy of 53% against rotavirus infections, 73% against rotavirus-related hospitalizations, and 74% against severe diarrhea episodes (38).

### Vaccination to Prevent Typhoid Fever

Typhoid fever is a life-threatening systemic disease caused by human adapted *Salmonella enterica* serovars, such as Typhi, Paratyphi A, Paratyphi B, and Paratyphi C (39, 40). These are Gram negative enterobacteria that infect humans by contamination of food and water supplies, causing disseminated infections that compromise internal organs, such as spleen, liver, bone marrow, and blood (39, 41). The incidence of these diseases is low in industrialized countries (less than 10 cases per 100,000) and high in developing countries, specifically in Asia and in Africa (more than 100 per 100,000) (42–44). Importantly, a significant increase in *S*. paratyphi A has been reported in the last years in Asian countries, reporting up to 44-fold increase in the period 2007–2013 in Cambodia (45). Currently, there are three licensed vaccines to prevent typhoid fever: The Vivotif^®^, Typbar^®^, and the Typhim V^®^ vaccines. The Vivotif^®^ is a live attenuated vaccine approved by the FDA for use in humans, based on the Ty21a strain, which was generated in the 1970 by chemical mutagenesis (46). This vaccine was previously produced and distributed by Crucell Switzerland It Ltd., but recently the American company PaxVax has acquired the license for this product. This vaccine is provided as a lyophilized formulation (in capsules) and used orally to promote mucosal immunity against these bacteria. A large clinical study performed in Chile showed that the rate of protection after three immunizations was 69% (47). In contrast, the Typhim Vi^®^ and Typbar^®^ are inactivated vaccines consisting of the Vi capsular polysaccharide, which are produced by Sanofi Pasteur and Bharat Biotech, respectively (48). The Typhim Vi^®^ vaccine is administered intramuscularly and confers an antibody-based protection (49). The rate of protection for this vaccine is close to 75% (50). Further, those vaccines do not confer cross-immune protection against *S*. Paratyphy A, for which does not exist a licensed vaccine available to prevent disease caused by this bacterium (51). Because of the immune memory conferred by both vaccines are very limited, their inclusion in immunization programs has not been recommended. However, the use of this vaccine has been encouraged by the WHO, especially when sanitation measures are threatened, for instance during natural disasters that impair the accessibility to clean water. Nevertheless, due to the growing emergence of antibiotic-resistant strains of *S*. Typhi in developing countries like India, the permanent use of these vaccines, as well as the generation of improved ones, would be highly appropriate to apply in their populations (52).

### Vaccination to Prevent Meningitis

Meningitis is an inflammation of the membranes covering the brain and spinal cord known as meninges, which can be caused by viral, bacterial or fungal infection, but also by due to non-infectious causes, as it has been reported (53). The main bacterial agents responsible for this disease are *S. pneumoniae*, Hib, and *N. meningitidis*, which could be prevented by available vaccines (54). Meningococcal disease has been reported worldwide, but largest epidemics have affected mainly sub-Saharan African countries, known as the “meningitis belt” having 430 million of high-risk population (53, 55).

According to the recent report in May 2017 by the CDC (56), there are two types of meningococcal vaccines available in the USA. The first vaccine is based on bacterial conjugates: Menactra^®^ and Menveo^®^, both conferring protection against A, C, W, and Y meningococcal serogroups. The second is a serogroup B recombinant meningococcal vaccine: Bexsero^®^ and Trumenba^®^. An additional vaccine, named MenAfriVac^®^ (produced by the Serum Institute of India Private Ltda.), confers protection against *N. meningitidis* serogroup A (Nm A), which is the most prevalent in the African “meningitis belt” (55). The MenAfriVac^®^ vaccine was a result of collaborative efforts between the WHO and the PATH in the Meningitis Vaccine Project, with the purpose of developing a vaccine against the specific agent affecting importantly the health of the African population, presenting a low-cost manufacturing and being independent of the cold chain distribution (57, 58). Since the national routine immunization strategic plan started in 2010, the incidence of Nm A meningitis fell from 0.27 per 100,000 in 2004–2010 to 0.02 per 100,000 in 2011–2013 (59). According to the recent WHO weekly record, 19 of the 26 countries belonged to the African “meningitis belt” have shown a sustained decreased incidence for Nm A cases, which means a reduction by at least 57% of the meningitis burden in that area (55). Also, clinical trials demonstrated that MenAfriVac^®^ decreases carriage rates in immunized populations and provides herd immunity probably because of the high antibody titers observed during the development and safety testing of the vaccine (60, 61). Due to the national immunization program for this vaccine was a success, Ghana and Sudan currently include the MenAfriVac^®^ in their routine immunization schedule (55). Despite the significant decrease in the prevalence on Nm A, it is important to highlight the necessity to continue with immunization programs to guarantee protection against different serogroups (62). Further, experts alert of the possible serogroup replacement, following application of massive immunization programs (63). In fact, in 2015 an epidemic with a novel strain of *N. meningitidis* serogroup C was reported in Niger and Nigeria. In addition, in 2016 the principal *N. meningitidis* serogroup W was found in Ghana and Togo, although with a low number of cases (55). For that reason, the continuous research in this area is a central challenge toward elimination of meningococcal meningitis epidemics in Africa.

## Vaccine Types, Manufacturing Procedures, and Current Research on Manufacturing Status

### Types of Vaccines and Manufacturing Procedures

Vaccines can be classified as live-attenuated, inactivated, subunits, recombinant, conjugated, toxoids, or DNA, according to the final preparation of the microorganism or antigen (64). Live-attenuated and inactivated microorganisms cover the major fraction of licensed vaccines for use in humans. Smallpox, BCG, yellow fever, polio, chickenpox, rotavirus, typhoid fever (Ty21a vaccine), and influenza are examples of licensed vaccines produced with live attenuated microorganisms (8, 65–67). On the other hand, examples of inactivated vaccines include those preventing plague, whooping cough, influenza, polio, typhoid fever (Vi capsular polysaccharide vaccine), and hepatitis A diseases (5, 49, 68–72). Only few vaccines are produced using recombinant technologies (hepatitis B virus, influenza, HPV) or *via* purification of partial components of a microorganism [*S*. Typhi Vi capsular polysaccharide, diphtheria, tetanus, pneumococcus, meningococcus, Hib, pertussis toxoid, and anthrax protective antigen (PA)] (73, 74). However, there has been an increased interest in the usage of these technologies in the past years (75).

There are different methods of vaccines production, which include isolation of microorganisms from either infected tissues (e.g., smallpox), bacteria growth in fermenters (e.g., vaccines for TB, typhoid fever, plague, whooping cough, diphtheria, tetanus, pneumococcus, meningococcus, pertussis, anthrax), isolation from virus grown in cell cultures (e.g., polio, chickenpox, rotavirus, hepatitis A virus (HAV), influenza) or isolation from virus grown in eggs (e.g., influenza, yellow fever). For the case of bacteria grown in fermenters, is not the microorganism itself that is used for the vaccine elaboration, rather some of its components from cell-free filtrates (e.g., vaccines for tetanus, pertussis, anthrax). For example, the anthrax vaccine adsorbed consists in the PA purified from filtrates by precipitation with alum, which also serves as an adjuvant (76).

An interesting change in the way of manufacturing has been recently carried out for influenza vaccines, which has been produced for more than 50 years in embryonated chicken eggs (77). However, GlaxoSmithKline (GSK) and Seqirus are currently producing influenza virus using cell culture technology in bioreactors (approved by the FDA in 2012) to generate new licensed influenza vaccines (78). Likewise, the Kaketsuken vaccine company is working on the development of a cell culture-based process, using the EB66 cell line, to elaborate a vaccine for pandemic flu, which is currently under clinical studies (79, 80). More recently, the Protein Sciences Corporation received approval for commercialization of a licensed novel influenza vaccine consisting of purified recombinant hemagglutinin antigens expressed in insect cell cultures (81). Similar efforts are in progress toward the development of cell culture-based yellow fever vaccines using Vero cell cultures in microcarriers (82). For anthrax, a plant-derived recombinant protective antigen has been developed as a vaccine, which is currently under evaluation in clinical trials (76, 83).

Thus, cell culture technologies, together with the enhancement of upstream and downstream processes, will bring production efficiencies to a next level as compared to the egg-based technology, and will increase manufacturing speed and capacities, thereby avoiding the shortage of these vaccines in the future (84, 85).

### Vaccine Research in the Industry versus the Academia

Vaccine portfolios in many pharmaceutical companies have decreased in the last decades due to the cost and time involved for vaccine development, which are much more costly and time consuming to develop than other drugs (86). However, pharmaceutical companies as well as academic institutions are continuously investing in vaccine research. For example, the number of vaccines in development has increased about twofold, according to a study comprising the 1995–2008 period in the USA (87). This fact can be explained, in part, by the advancement of alternative technologies, such as baculovirus-based recombinant vaccines, virus-like particles, viral vectors and RNA or DNA vaccines (74, 88–93). Moreover, with a world population projected to be of 10 billion by 2050, a 90% of it is estimated to live in developing countries (United Nations projection) (94). Thus, the subsequent increase in the vaccine market from USD 25 billion by today to USD 100 billion by 2025, will continue to encourage vaccine research and development (95).

Many research groups in academic institutions have made considerable efforts on vaccine discovery and research, but only few of them have been able to move forward into the development vaccine process. A reduced technology transfer efficiency may be due to difficulties on establishing private-academy license agreements (LA) (96). Indeed, Public-Private-Partnerships (PPP) has shown to be relevant for some vaccine developments, such as for the prototype of HIV vaccine (97). Thus, these LA and PPP enable the implementation of new and improved vaccines in high-tech centers before a product is transferred into the market. Another factor is the requirement of facilities with Good Manufacturing Practicing (GMP) certification and high-quality personnel to develop vaccine production processes. The staff capacities and facilities to investigate, develop and manufacture vaccines are key to respond rapidly to the global emergencies, such as the recent Ebola outbreak (98).

The increase of vaccine manufacturers has impacted on the global market, allowing to lower the prices of vaccines and to improve the global demand. Further, partnerships, such as GAVI Alliance, UNICEF, and the WHO have also been key for enhancing that kind of vaccine production in developing countries (99). As example, the new vaccine manufacturing countries such Brazil, the Russian Federation, India, China, and South Africa (known as BRICS) play a substantial and increasing role in the global vaccine market. These countries not only produce traditional vaccines at competitive low costs and under the WHO-prequalified standards, but they also generate innovative products due to current strategic alliances with multinational corporations (99, 100). The most successful case of this strategic alliance, is the Bio-Manguinho plant in Brazil, that will be producing an affordable measles and rubella vaccine with the support of the Bill & Melinda Gates Foundation together with the Brazilian Ministry of Health (100). An arising number of pharmaceuticals along with the NIH are interested in enlarging the number of vaccines manufactured in those institutes, which in turn involved discussion of the agreement of the Trade Related Aspects of Intellectual Property Rights.

## Diversity of Immunization Programs Worldwide: Regional Examples and the Gap Between Industrialized and Developing Countries

Worldwide, the diversity in national immunization programs is extensive, therefore the list of vaccines included and distributed in each country shows significant differences (Figure 1). Furthermore, the vaccination plan for the USA might even be different depending on the state, while in Europe the immunization plans have significant differences among the countries belonging to the European Union (Figure 1). On the other hand, there are variations in the financing mechanisms for vaccine production within Europe. For instance, the National Health System funds the rotavirus vaccine in Germany, but not in Spain. Other vaccines, such as the live attenuated Japanese encephalitis, cholera, and yellow fever vaccines are recommended only in some Asian countries, such as in India and in Thailand. Furthermore, meningococcal C conjugate vaccines are included in the National Health System of Australia, Chile, and Spain, but not in those of Asian countries like in India. Another example of diversity on immunization schedule is the BCG vaccine against TB. This vaccine is being administered only in some countries in Europe, Asia, Africa, and South America, but it is not administered in industrialized countries such as in the USA (14). Table 1 summarizes the differences of the immunization programs between seven countries, including industrialized and developing countries (101, 102).

**Table 1:** National immunization programs of seven countries.

	BCG	HepB	Polio	DTaP	MMR	HPV	Hib	Pneumococcal	Rotavirus	JE
USA		2, 4, and 6 months old	2, 4, 6 months and 11 years old	2, 4, and 6 months old	12 months old	>11 years old	2, 4, and 6 and >12 months old	2, 4, and 6 and >12 months old	2, 4, and 6 months old	
Chile^a^	Newborn	2, 4, and 6 months old	2, 4, 6 months and 12–13 years old	2, 4, and 6 months old	12 months old	10 years old	2, 4, and 6 months old	12 months old		
Germany		2, 3, 4, 11–14 months old	2, 3, 4, 11–14 months and 5–6 and 9–11 years old	2, 3, 4, and from 11 to 14 months years old	11–14 and 15–23 months years old	9–14 years old	2, 4, 4, and 12–14 months old	2, 4, and 11–14 months old	6 weeks, 2 and 4 months old	
Spain		2, 4, 6 months old	2, 4, 6, and 18 months old	2, 4, 6, and 18 months old, and 6 years old	12 months and 3–4 years old	12–14 years old	2, 4, 6, and 18 months old	2, 4, and 11 months old^b^		
China^a^	Newborn	Newborn, 1 and 6 months old	2, 3, 4 months, and 4 years old	3, 4, 5, and from 18 to 24 months years old	18–24 months old					8 months and 6 years old
India^a^	Newborn	Newborn	6, 10, 14 weeks, and 16–24 months old	6, 10, 14 weeks, and 16–24 months old	9, 16–24 months^c^					9, 16–24 months old
South Africa^a^	Newborn	6, 10, 14 weeks and, 18 months old	Newborn, 6 weeks	6, 10, 14 weeks, and 18 months old	9 and 18 months^c^		6, 10, 14 weeks, and 18 months old	6, 14 weeks, and 9 months old	6 and 14 weeks old	

*Developed and developing countries were selected according to their geographical area and income. Orange: not funded by the public health system. Blue: funded by the public health system*.

*^a^Developing countries*.

*^b^Depends on the region, this vaccine is included in the public health system*.

*^c^Only vaccine against measles*.

*BCG, bacille Calmette–Guerin; HepB, hepatitis B virus; DTaP, diphtheria, tetanus, and pertussis; MMR, mumps, measles, and rubella; Hib, Haemophilus influenzae type B; HPV, human papillomavirus; JE, Japanese encephalitis live vaccine*.

Germany is an example where vaccination is mostly voluntary with a reduced role of the state in the implementation of vaccination programs. Around 90% of the vaccines are given by private physicians and only the remaining small fraction of the vaccines is given by public institutions, schools or daycare centers (103). Massive school immunization programs are not mandatory, but the immunization status is checked at schools. This information is collected and documented by the Robert Koch Institute. The Berlin measles outbreak of 2015 and the death of a non-vaccinated infant raised the discussion as to whether vaccination in Germany must be mandatory (104). This discussion has been intensified considering that the Europe is confronting the largest immigration since the World War II. The collapse of national immunization programs in the countries undergoing political turmoil has led to children-disease outbreaks, which could have been prevented by vaccination. Moreover, refugees are susceptible to diseases due to overcrowding, physical and psychological stress, malnutrition and low availability of sanitary systems. These health aspects and conditions constitute a serious threat to immigrants, as well as to international programs aimed at eradicating vaccine-preventable diseases. Recent studies of measles, mumps, rubella, and varicella seroprevalence in refugees in Germany have shown satisfactory immunity in adults but low seroprevalence in children, suggesting thorough and prompt vaccination of children entering Europe (105). The opposite has been found for hepatitis A immunity in refugees in Germany, where the high rate of HAV protection supports the thesis that the probability of large HAV outbreaks in current German refugee centers is low (71). Nevertheless, vaccination of refugees against HAV is highly recommended.

The immunization programs in the USA follow the CDC guidelines (106). In this country, as mentioned earlier, vaccine coverage differs widely among states, varying for instance with ≥2 doses of HAV from 41.2% in Mississippi to 72.8% in Nebraska (107). Recent nonmedical exemptions in immunization laws have prompted serious concerns about potential vaccine coverage weakening. However, after the recent outbreaks of vaccine-preventable diseases mandatory immunizations at entry-schools and primary care facilities have emerged. Indeed, those states that allow exemptions, including religious and philosophical reasons, have shown a significantly higher incidence of vaccine-preventable diseases, as compared to those states allowing less exceptions for vaccination (108). Interestingly, the coverage of vaccines in the USA will depend on the insurance plan of each individual. Accordingly to the CDC, the coverage of children aged 19–35 months was lower in those children uninsured or covered by public insurance programs, such as Medicaid, as compared to private insurance-covered kids (107). However, some the USA vaccine manufacturers and the National Vaccine Programs offer help to those people who cannot afford some vaccines, such as the one for HPV. Importantly, up to 32.9% of children of 19–35 months of age in the USA live below poverty level and can fail to receive all the required vaccines (107). To overcome this problem, the Vaccines for Children Program in the US offers free vaccines to children living in poverty (107).

In South America, the Pan-American Health Organization (OPS) provides a caring cooperation system, named the “Fondo Rotatorio,” designed to obtain the vaccines recommended by the WHO at low prices (109). As for the case of Chile, the Public Health Institute and the Ministry of Health direct the Chilean National Immunization Program (CNIP) following international recommendations. Vaccines included in the CNIP are funded by the government and given to hospitals, family health centers and some schools in Chile. The introduction of the latest vaccines in the CNIP has significantly reduced the incidence of certain diseases, such as bacteria-caused pneumonia and cervical cancer. One example is the 10-valent pneumococcal vaccine, which was introduced in January 2011 and thereafter, the number of hospitalizations due to pneumonia were successfully reduced (110). Such effectiveness of the 10-valent pneumococcal vaccine has also been demonstrated in other South-American countries (111). In 2015, the Chilean government supported the introduction of the HPV vaccine in the CNIP and thereby, most of 9–10 years old girls have been vaccinated since then as a program to prevent cervical cancer.

**Figure 2:**
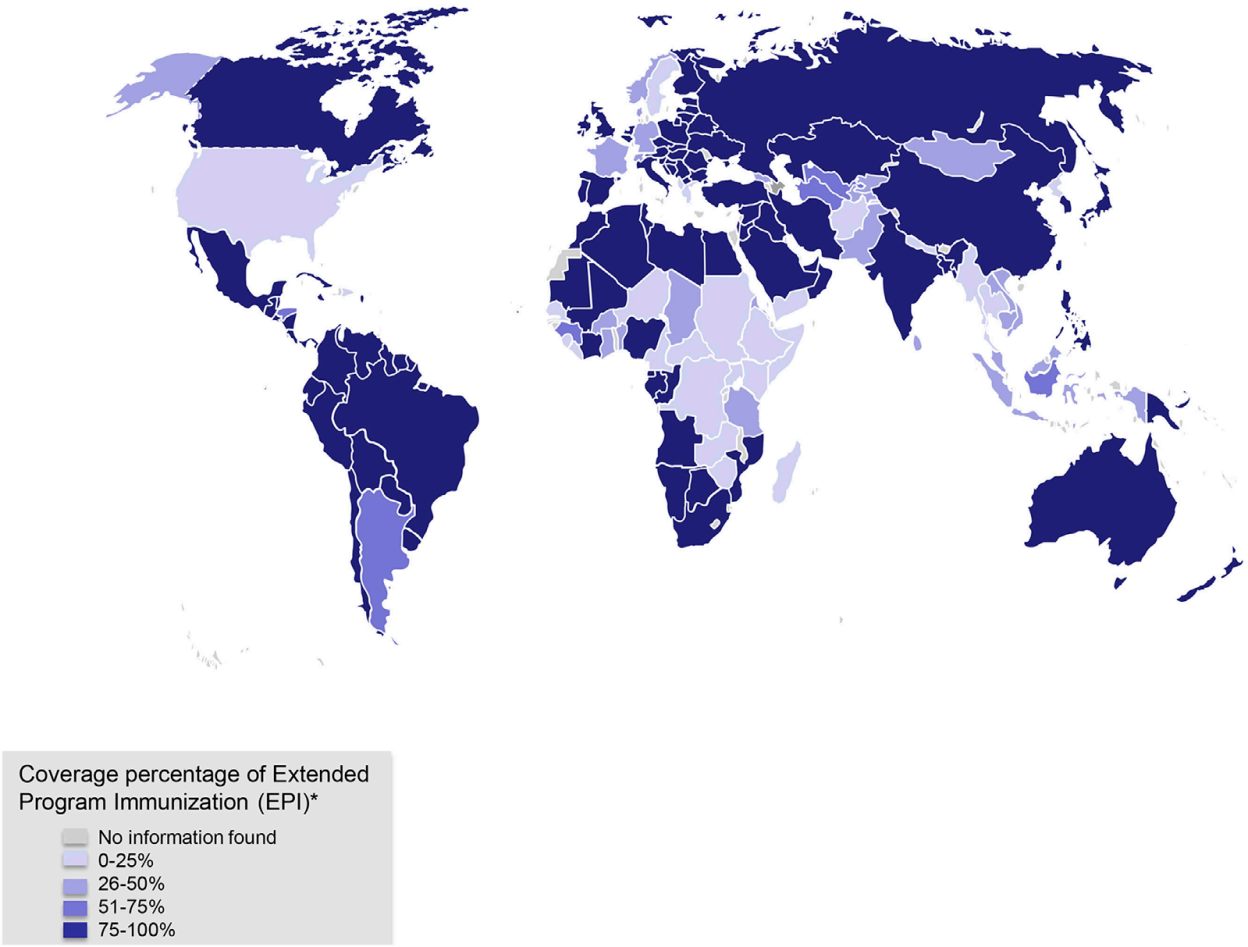
Coverage for Extended Program of Immunization (EPI). *EPIs include those against tuberculosis, diphtheria, tetanus, and pertussis (DTP), polio, and measles, as well as those protecting newborn children and their mothers against tetanus by vaccination of pregnant women. Data obtained from WHO/UNICEF reports 2007 and 2014 reports.

Thus, each country has its own national immunization program (112), which in most cases includes vaccines that are sponsored by their public health systems reaching different levels of coverage (Figure 2). Nevertheless, many developing countries have difficulties to finance all the vaccines recommended by the WHO. As a result, different organizations have arisen to provide economical support to the developing countries requiring vaccines. For instance, the Global Vaccine Activation Plan (GVAP) has established itself the goal of reducing some vaccine-preventable diseases by 2020 (113). Moreover, most traditional vaccines are sold at lower prices to organizations, such as UNICEF and the Pan-American Health Organization to reach developing countries (14). Although global coverage has improved, in countries such as India, Nigeria, Pakistan and Indonesia, a low immunization coverage still exist (113). It is noteworthy that 35 of the 45 classified as lower-middle income countries by the World Bank Classification are not being supported by GVAP Alliance, thereby these countries are struggling to reach underused and new vaccines to immunize their children (14). Also, one of the GVAP goals was to eliminate the maternal and neonatal tetanus, measles, and rubella in 2014, but unfortunately this goal was not achieved (113). One of the main reasons for this failure has been the unstable political situation in some countries and the inefficient introduction of these vaccines in national immunization programs (113). Therefore, economical gaps still remain between industrialized and developing countries to accomplish efficient immunization programs for their children. With globalization, leading to increased and fast movements of goods and people traveling to all remote areas in the world, these differences in health protection can be a risk for outbreaks, epidemics or even worse, pandemics. Importantly, several organizations including the GAVI Alliance, UNICEF, the Bill & Melinda Gates Foundation, the United States National Institute of Allergies and Infectious Diseases, the WHO, together with governments and other institutions support the goals of the GVAP to reduce some vaccine-preventable diseases by 2020 (113).

## Vaccine Manufacture and Distribution: Status of Academic, Public, and Private Manufacturing Companies

### Vaccine Production and Distribution

Although mainly private pharmaceutical companies have engaged in vaccine manufacturing and distribution, there are also successful efforts made by academic or public institutions to achieve this goal (Table 2). Vaccine manufacturing requires specific and expensive facilities with high scale production, and quality standards to ensure consistency and controlled elaboration of these products. This is typically achieved following the guidelines of the current (c) GMP in compliance with the local regulatory authorities. Therefore, most of the countries have contract agreements with specific cGMP-certified manufacturers to purchase the vaccines required for their populations. For example, the private sector is in charge of the 5–10% of the vaccines market in Asia (114).

**Table 2:** List of vaccine manufacturing centers companies of the countries reviewed in this work.

Name of company institute	Country	Vaccines manufactured
Statens Serum Institute	Denmark	BCG
GlaxoSmithKline	UK, Italy	Meningococcal, tetanus toxoid, acelullar pertussis, reduced diphtheria toxoid, HPV, HepB, influenza, HepA, Hib, meningococcal, rabies, rotavirus
Seqirus	UK	Difteria and tetanus, cholera, HPV, HepB, JE, meningococcal, MMR, influenza, pneumococcal, rabies, rotavirus, HepA
Sanofi	France	Cholera, diphtheria, pertussis and tetanus, Hib, meningococcal, BCG, typhoid fever, dengue, HepA, HepB, influenza, JE, polio, rabies, yellow fever
Immunobiological Technology Guinhos (Bio-Manguinhos/Fiocruz)	Brazil	Yellow fever, polio, meningitis A, MMR, rotavirus, Hib, pneumococo
Butantan Institute	Brazil	Diphtheria toxoid and tetanus toxoid, DTP-whole cell, influenza, hemorrhagic fever/dengue, HepB, rabies
Sinergium Biotech	Argentina	Influenza, pneumococcal, HPV
ANLIS	Argentina	BCG, rabies, tetanus toxoid, yellow fever
Fundaçao Ataulpho de Paiva	Brazil	BCG
Birmex	Mexico	Diphtheria toxoid and tetanus toxoid, polio
Pfizer	US	Meningococcal, pneumococcal
Merck	US	BCG, HPV, Hib, MMR, pneumococcal, HepB, rotavirus, HepA, varicella
Serum Institute of India	India	DTP, MMR, Hib, meningococcal, influenza, BCG, HepB, Polio
Bharat Biotech International	India	Rotavirus, Hib, polio, DTP, influenza, rabies, typhoid
Kaketsukken	Japan	DTP, influenza, JE, HepB, rabies
China National Biotec Group Company Limited	China	DTP, BCG, influenza, Hib, hemorrhagic fever, JE, meningococcal, MMR, polio, rabies, rotavirus, varicella, yellow fever
BioNet	Thailand	Acelullar pertussis
Biofarma	Indonesia	BCG, diphtheria, tetanus, DTP-HepB-Hib, HepB, measles, polio
GreenSignal Bio Pharma Limited	India	BCG
IVAC	Vietnam	BCG, DTP
Pasteur Institute of Iran	Iran	BCG, HepB
Queen Saovabha Memorial Institute	Thailand	BCG, rabies
Vabiotec	Vietnam	Cholera
Vacsera	Egypt	Cholera, diphtheria, tetanus
Eubiologics	South Korea	Cholera, diphtheria, tetanus
Biological E. Limited	India	Diphtheria, tetanus, DTP, HepB, Hib, HepB, JE, tetanus toxoid
Instituto Finlay de Vacunas	Cuba	Tetanus toxoid, DTP
Indian Immunological Ltd.	India	Diphtheria toxoid and Tenatus toxoid, DTP, rabies
SK Chemicals	Korea	HepB, influenza, tetanus-diphtheria
Razi	Irán	DTP, MMR, polio
Haffkine	India	Polio
TiantianBio	China	Rubeolla
Torlak Institute	Serbia	BCG, diphtheria, tetanus
Biovac	South Africa	BCG

*BCG, bacille Calmette–Guerin; HepB, hepatitis B virus; DTP, diphtheria, tetanus, and pertussis; MMR, mumps, measles, and rubella; Hib, Haemophilus influenzae type B; HPV, human papillomavirus; JE, Japanese encephalitis live vaccine*.

The USA is one example of a country, in which both private and public sectors provide vaccines for their population (115). This is an advantage, because the public health system can choose from different sources and prices. The main pharmaceutical companies that produce and distribute vaccines around the world include GSK, the United Kingdom; Pfizer, the USA; Sanofi Pasteur, France; Merck & Co., the USA; Roche, France; Seqirus, Australia; Valneva SE, France (Table 2). In addition, emerging pharmaceutical companies, such as Astellas Pharma, Japan; Takeda, Japan, and AstraZeneca, United Kingdom currently invest in vaccine R&D. Other international companies, including the Serum Institute of India and the Bharat Biotech International supply vaccines to countries without local vaccine manufacture facility, such as Chile. Particularly, the Serum Institute of India is a state-owned vaccine manufacturing center that produces most of the vaccines recommended by the WHO including BCG, polio, Hib, DTaP, and MMR. Similarly, national public enterprises, including the Immunobiological Technology Guinhos (Bio-Manguinhos/Fiocruz) and the Butantan Institute supply most of the vaccines in Brazil (Table 2). Importantly, the two institutions previously mentioned supply about up to 83% of the Brazilian National Immunization Program demand, thereby reaching up to 179,855,000 national doses (116). A different situation can be found in Germany, where most of the vaccines are purchased from the private sector (90%) and 90% of them are financed by statutory insurance policies (117). The government provides the rest of the vaccines as part of special immunization programs. Recent studies have shown that no more than 0.47 and 0.25% of the German and Spanish healthcare budget, respectively, are addressed to vaccine production (117).

Due to the problems stated above, in the year 2000 an organization aimed to create alliances of vaccine manufacturers in developing countries was established. This organization, known as the Developing Countries Vaccine Manufactures Network (DCVMN), includes near 50 vaccine manufacturers in 17 developing countries in Latin America, Africa, the Middle East, and Asia (118–120). The companies that are members of this organization produce more than 40 different vaccines, including the ones recommended by the WHO including BCG, polio, Hib, DTaP, and MMR (Table 2) (118, 120). Although the DCVMN main goal is to provide a high quality (cGMP compliant) and sustainable supply of vaccines for developing countries, there are still not enough to provide the increasing demand of vaccines.

### Vaccine Shortages

The coverage of the national immunization programs relies on the available supply of vaccines. Several countries have experienced vaccine shortages at some point, which have included BCG, Hib, DTaP, pneumococcal conjugate, MMR, meningococcal, yellow fever, and influenza vaccines (121, 122). As an example, Sanofi Pasteur, one of the major producers of BCG, the current vaccine for TB, experienced significant manufacturing problems during 2012 and 2014. As a result, distribution of this vaccine was seriously compromised in several countries (123). Indeed, approximately 16.5 million doses shortfall of BCG occurred at the end of 2015 was estimated, using mathematical models, to be associated with 7,433 excess of TB deaths worldwide (124). In 2015, short supplies for the meningococcal vaccine worldwide threatened the health of the population in Nigeria, a place where an important epidemic of meningitis took place (125). An additional example is the Hib boost vaccine, for which doses were not available in the USA from December 2007 to September 2009 (122). Moreover, several physicians have reported shortages of influenza vaccines, especially for high-risk populations in the USA during the years 2004–2005 (126). Further, Africa and the USA have also experienced shortages for the yellow fever vaccine during the last 2 years (127–129). Similarly, significant shortages of the pneumococcal conjugate vaccine occurred during the period 2003–2004, causing an important decrease of 10.6% of the coverage of >4 doses of the seven-valent pneumococcal conjugate vaccine in 16-month-old children (130). Likewise, such shortage issues have prompted the concern of elaborating protocols for ensuring availability of those vaccines for at least the high-risk populations (131). Because the pandemic of influenza is highly extensive, the demand for this vaccine worldwide is very high, causing sometimes problems of vaccine shortage (132, 133). This situation is particularly dramatic when pandemics on influenza arise, such as the H1N1 in 2009 (134).

Different reasons can explain disruptions of the vaccine supplies, such as vaccines that leave the market, problems in the production, loss of the GMP in manufacturing centers/companies, and changes in the formulation of vaccines (135). An important correlation is that fewer vaccine manufacture suppliers exist for one vaccine the larger the impact of supply shortage can have on the population (135). To solve the vaccine shortage in case of epidemics, global vaccine stockpiles have been established for vaccines, including smallpox, meningococcal, yellow fever, oral cholera, and pandemic influenza vaccines (136). Moreover, the challenge for institutions, such as the Brazilian government, is to make investments for local vaccine development and manufacturing to avoid international dependency and the threat of shortage (116).

## Global Emerging Diseases and Antibiotic Resistance

The Ebola, Zika, and influenza virus pandemics are examples of worldwide emergencies that have recently affected various regions of the planet. In 2009, the H1N1 influenza pandemic resulted in the highest number of cases in Mexico (134). In April of 2009, the first cases with severe respiratory disease started to be concentrated in the Federal District of Mexico’s most populated area. The Mexico’s National Institute of Respiratory Disease struggled with such situation to contain the propagation of the influenza virus (137). Months later, the H1N1 virus was spread to over 213 countries causing 16,226 deaths and the WHO declared it to be the first flu outbreak in the last 41 years (138). The H1N1 2009 pandemic was identified as a new influenza A subtype of swine origin, and consequently, at that moment no vaccines were available. After that outbreak, a vaccine was rapidly developed, include the 2009 H1N1 influenza virus antigen in order to protect against that virus (139). However, if new mutations arise resulting in a new pandemic subtype, then the available vaccine will be useless and again no vaccine will be accessible to protect against a potential new virulent strain with a high rate of mortality, such as seen with the previous H1N1 influenza A virus pandemics.

In 2014, West Africa experienced a devastating outbreak of Ebola and multiple countries were affected. In response to that situation, several countries and institutions such as the WHO and the CDC activated emergency operations to control the situation (98). Although the end of transmission of Ebola was reported in Liberia and in Guinea, still the WHO in Guinea, Liberia and Sierra Leone has still reported a total of 28,616 Ebola cases, with 11,310 deaths (140). Ebola virus is associated with hemorrhagic fever and is transmitted by corporal fluids. No vaccine or treatment is available for this virus; thereby efforts in that situation were to limit transmission of the disease.

On the other hand, according to the CDC, most of the Zika virus cases have been reported in many countries of South America, Africa, Asia, and the USA (141). This virus is transmitted by a mosquito-borne (*Aedes aegypti*) and symptoms include mild fever, headache, arthralgia, myalgia, non-purulent conjunctivitis, and a pruritic maculopapular rash (142). However, the most concerning effect that has been associated with Zika virus is the prenatal microcephaly (143).

According to the WHO-vaccine pipeline tracker, vaccines against AIDS, malaria, enteric pathogens, including human norovirus, the respiratory syncytial virus, Zika virus, Dengue virus, and pulmonary TB are in different stages of development. Some of these diseases, such as AIDS or pulmonary TB have been a concerning problem, since for several years have not been obtained a definitive cure or an efficient vaccine to prevent them. In addition, other diseases, such as the ones caused by the Zika virus, have had emergency problems that have required a rapid response. One prompt response strategy for the past Ebola outbreaks has been the use of anti-Ebola antibodies from the blood of disease survivors. Therefore, strategies with monoclonal antibodies to treat Ebola are currently being studied (144). Moreover, research on nanoparticles, adenovirus-based, modified Vaccinia Ankara-based, and recombinant-rabies vaccines against Ebola are ongoing, even in phase I, II and III of clinical trials (98, 145). From Ebola vaccines in clinical trials so far, the most advanced one is a recombinant vesicular stomatitis virus–Zaire Ebola virus (rVSV-ZEBOV) vaccine that has been licensed to Merck and recently, showed to be effective in susceptible individuals (146). On the other hand, strategies such as adenovirus-based recombinant vaccines and cell culture-derived inactivated vaccines using BHK and Vero cells are under research for Zika virus vaccine development (147). Despite the research ongoing about Zika and Ebola viruses, or other common and fastidious viruses such as respiratory syncytial virus and human norovirus, no vaccines or efficient treatment are still available. Thus, high technology centers are urgently needed to provide a solution to these problems and offer a rapid response to global health emergency states.

As emerging diseases, microorganisms with multiple resistances to antimicrobial agents have been reported in the past years. Bacteria resistance to the available antimicrobial agents, such as *Klebsiella pneumoniae, Staphylococcus aureus, Escherichia coli*, and *Mycobacterium tuberculosis* have alarmed health care worldwide for their resistance to antimicrobial agents (148–151). Furthermore, availability of an effective therapy for patients infected with those microorganisms is limited and more research and development is needed (152). Despite policies concerning the use of antimicrobials and the development of new drugs, it is urgent to increase the vaccine manufacturing capacity to prevent the spreading of these infections with multiple antibiotic resistance (153).

## Concluding Remarks

There is no doubt that many diseases have been prevented due to the implementation of extensive vaccination programs. Domestic health public systems worldwide are committed to increase vaccination coverage for the population through national immunization programs. Thus, the WHO recommends to immunize children with BCG, DTaP, MMR, and vaccines to prevent hepatitis B, poliovirus, Hib, several serotypes of *S. pneumoniae*, rotavirus, and HPV. However, not all these vaccines are included in the national immunization programs of most countries. Not only the problem is the inclusion of some vaccines in local programs of immunizations but also the cost associated with its production, implementation, and delivery are part of the barriers. In this line, it is important to highlight the effort of some organizations such the WHO, the PATH, the GAVI Alliance, the UNICEF, the Bill & Melinda Gates Foundation, among others, to include as much population as possible in these immunization global strategies. Furthermore, shortages around the world have taken places during the past years, which have underscored the necessity to improving the capacities and infrastructure to produce and distribute vaccines. It is important to underscore the role played by new countries manufacturing vaccines, which include Brazil, the Russian Federation, India, China and South Africa (a group known as BRICS). Such local production has contributed to ensuring access to traditional vaccines and to maintaining the stability of immunization programs in developing countries. Also, an important gap between industrialized and developing countries prevails in this field. Further, Ebola, Zika, influenza virus pandemics, and antimicrobial resistance have raised alarms, questioning whether we are prepared to control rapidly and efficiently viral pandemics worldwide.

## Author Contributions

ERJ, FT, NMD, and AK wrote the manuscript; ML, LC, SB, CR, and YG reviewed the manuscript; and AK reviewed and approved the version to be published. All authors listed have made substantial and intellectual contribution to the work.

## Conflict of Interest Statement

The authors declare that the research was conducted in the absence of any commercial or financial relationships that could be construed as a potential conflict of interest.
